# Up-Regulation of CYP2C19 Expression by BuChang NaoXinTong via PXR Activation in HepG2 Cells

**DOI:** 10.1371/journal.pone.0160285

**Published:** 2016-07-28

**Authors:** Hong Sun, Xiao-Ya Lou, Xiao-Ying Wu, Huan Wang, Qiang Qu, Shen-Lan Tan, Jun-Shan Ruan, Jian Qu, Hui Chen

**Affiliations:** 1 Department of Pharmacy, Provincial Clinical College of Fujian Medical University, Fujian Provincial Hospital, Fuzhou, Fujan, P. R. China; 2 Department of Clinical Pharmacology, Xiangya Hospital and Institute of Clinical Pharmacology, Central South University, Hunan Key Laboratory of Pharmacogenetics, Changsha, Hunan, P. R. China; 3 Hypertension Laboratory, Provincial Clinical College of Fujian Medical University, Fujian Provincial Cardiovascular Disease Institute, Fujian Provincial Hospital, Fuzhou, Fujian, P. R. China; 4 Department of Pharmacy, Xiangya Hospital, Central South University, Changsha, Hunan, P. R. China; 5 Department of Pharmacy, the Second Xiangya Hospital, Central South University; Institute of Clinical Pharmacy, Central South University, Changsha, Hunan, P. R. China; Hokkaido Daigaku, JAPAN

## Abstract

**Background:**

Cytochrome P450 2C19 (CYP2C19) is an important drug-metabolizing enzyme (DME), which is responsible for the biotransformation of several kinds of drugs such as proton pump inhibitors, platelet aggregation inhibitors and antidepressants. Previous studies showed that Buchang NaoXinTong capsules (NXT) increased the CYP2C19 metabolic activity *in vitro* and enhanced the antiplatelet effect of clopidogrel *in vivo*. However, the underlying molecular mechanism remained unclear. In the present study, we examined whether Pregnane X receptor (PXR) plays a role in NXT-mediated regulation of CYP2C19 expression.

**Methods:**

We applied luciferase assays, real-time quantitative PCR (qPCR), Western blotting and cell-based analysis of metabolic activity experiments to investigate the NXT regulatory effects on the CYP2C19 promoter activity, the mRNA/ protein expression and the metabolic activity.

**Results:**

Our results demonstrated that NXT significantly increased the CYP2C19 promoter activity when co-transfected with PXR in HepG2 cells. Mutations in PXR responsive element abolished the NXT inductive effects on the CYP2C19 promoter transcription. Additionally, NXT incubation (150 and 250μg/mL) also markedly up-regulated endogenous CYP2C19 mRNA and protein levels in PXR-transfected HepG2 cells. Correspondingly, NXT leaded to a significant enhancement of the CYP2C19 catalytic activity in PXR-transfected HepG2 cells.

**Conclusion:**

In summary, this is the first study to suggest that NXT could induce CYP2C19 expression via PXR activation.

## Introduction

Cytochrome P450 2C19 (CYP2C19) is one of important drug-metabolizing enzymes (DMEs), which are involved in the biotransformation of many drugs such as proton pump inhibitors, platelet aggregation inhibitors and antidepressants[1]. Growing evidences suggested that CYP2C19 expression is susceptible to many drugs or herbs, leading to some unpredictable drug-drug interaction (DDI), which may cause adverse effects or failure in the therapy [2–5].

BuChang NaoXinTong (NXT) capsule is an approved Chinese traditional medicine, which is widely used to treat stroke and cardiovascular diseases in China[6]. It contains 16 different traditional Chinese medicines and herbs as follows: *Astragalus membranaceus* (Fisch.) Bunge (*Astragalus propinquus* Schischkin), roots, dried; *Salvia miltiorrhiza* Bunge., roots and rhizomes,dried; *Ligusticum chuanxiong* S.H.Qiu, Y.Q.Zeng, K.Y.Pan, Y.C.Tang & J.M.Xu, roots and rhizomes, dried; *Angelica sinensis* (Oliv.) Diels., roots, dried; *Prunus persica* (L.) Batsch., seeds, dried; *Boswellia serrata* Roxb. ex Colebr., resin; *Commiphora myrrha* (Nees) Engl., resin; *Morus alba* var. *tatarica* (L.) Loudon., twigs, dried; *Paeonia veitchii* Lynch, Roots,dried; *Carthamus tinctorius* var. *albus* Alef., flowers, dried; *Spatholobus suberectus* Dunn., cane, dried; *Achyranthes bidentata* Blume., roots, dried; *Cinnamomum cassia* (L.) J.Presl., twigs, dried; *Pheretima aspergillum* (E. Perrier)., body, dried; *Buthus martensii* Karsch., body, dried; *Hirudo nipponica* Whitman., body,dried.[7]. Haiyu X *et*. *al* characterized and identified a total of 178 components in BuChang NXT, including 21 flavones and 6 flavone glycosides, 18 phenanthraquinones, and 22 terpenoids[8]. Our previous *in vitro* study indicated that NXT increased the CYP2C19 catalytic activities[9]. In addition, we also demonstrated that adjunctive NXT enhanced the antiplatelet effect of clopidogrel both in the volunteers and the patients undergoing percutaneous coronary intervention (PCI) with the CYP2C19*2 polymorphism[10, 11]. However, the underlying mechanism is not understood yet.

Pregnane X receptor (PXR, NR1I2) is a member of the nuclear receptor superfamily. It is one of key transcriptional factors responsible for regulating gene expression of several DMEs and drug transporters such as CYP3A4, CYP2B6[12–14]. An earlier study reported that rifampicin, a typical PXR ligand, induced CYP2C19 expression in human primary hepatocytes[4, 15, 16]. Chen *et al*. confirmed the observation and they identified that PXR stimulated the CYP2C19 transcription by directly binding to its promoter[15]. On the other hand, some active constituents of NXT ingredients could act as the PXR ligands, for example, tanshinone IIA and ligusticum were reported to enhance the CYP3A4 expression via the PXR pathway[13, 17]. Therefore, in our present study, we examined whether NXT could modulate CYP2C19 expression at the transcriptional level via activating PXR.

## Materials and Methods

### Materials

BuChang NXT capsules (Compilation of The National Standard of Chinese Traditional Medicine no. WS-10001 (ZD-0001)-2002; Med-drug Permit no. Z20025001;Pharmacopoeia of The People’s Rrepublic of China (2015, volume 4, page1379–1380)) were supplied by the Buchang Pharmaceutical Co. Ltd. The ingredients of BuChang NXT include *Astragalus membranaceus* (Fisch.) Bunge (*Astragalus propinquus* Schischkin), roots, dried; *Salvia miltiorrhiza* Bunge., roots and rhizomes,dried; *Ligusticum chuanxiong* S.H.Qiu, Y.Q.Zeng, K.Y.Pan, Y.C.Tang & J.M.Xu, roots and rhizomes, dried; *Angelica sinensis* (Oliv.) Diels., roots, dried; *Prunus persica* (L.) Batsch., seeds, dried; *Boswellia serrata* Roxb. ex Colebr., resin; *Commiphora myrrha* (Nees) Engl., resin; *Morus alba* var. *tatarica* (L.) Loudon., twigs, dried; *Paeonia veitchii* Lynch, Roots,dried; *Carthamus tinctorius* var. *albus* Alef., flowers, dried; *Spatholobus suberectus* Dunn., cane, dried; *Achyranthes bidentata* Blume., roots, dried; *Cinnamomum cassia* (L.) J.Presl., twigs, dried; *Pheretima aspergillum* (E. Perrier)., dried; *Buthus martensii* Karsch., dried; *Hirudo nipponica* Whitman., dried. BuChang NXT was flayed, triturated, quantified, and then was dissolved in dimethylsulfoxide (DMSO) (Sigma, USA)[9, 18]. Rifampicin was purchased from Sigma (USA); Dulbecco’s modified Eagle’s media (DMEM) and Fetal bovine serum (FBS) were provided by Gibco (USA); Trizol reagent was obtained form Invitrogen (USA); the first strand cDNA synthesis Kit and SYBR Green qPCR SuperMix were purchased from TaKaRa (Japan). Anti-CYP2C19 (Santa Cruz, USA), anti-β-actin (Sigma, USA) primary antibodies and HRP-conjugated second antibodies (Santa Cruz, USA) were used in this study. All other chemicals used in the study were commercially available and of reagent grade.

### Cell culture and treatments

HepG2 cells were originally obtained from America Type Culture Collection (ATCC; Manassas, USA) and cultured in DMEM with 10% FBS, 100U/mL penicillin and 100μg /ml streptomycin at 37°C in a humidified atmosphere containing 5% CO_2_.

NXT and rifampicin were dissolved in DMSO. For the treatment, HepG2 cells were seeded in 6-well plates and transiently transfected with pcDNA3.1-PXR or pcDNA3.1 plasmid for 24h (HepG2-PXR vs. mock cells, respectively) using Lipofectamine 2000 (Invitrogen; USA) according to the manufacturer’s instructions; and then incubated with different concentrations of NXT (150 and 250μg/mL) or 10μM rifampicin (positive control) for another 24 hours. The cells treated with 0.1% DMSO was used as control group. PcDNA3.1-PXR and pcDNA3.1 plasmids used in the experiments were a generous gift from Prof. Tu jiang-hua (Centre South University, China).

### Construction of *CYP2C19* reporter gene plasmid and luciferase assays

A 2286bp-length fragment of the *CYP2C19* 5’flanking promoter region (from -2102~ +186bp, using ATG as +1) was amplified using human genomic DNA as the template and this PCR production was cloned into pGL4.17-basic luciferase reporter plasmid (Promega; USA) between KpnI and XhoI enzyme sites to construct wild-type *CYP2C19* reporter gene vector (2C19-Wt). Primers used for 2C19-Wt amplification were as follows: F: 5’-GGTACCGGAGGAGCAGAACTGGAACAC-3’; R: 5’-CTCGAGGAAGGAGCATACTTACATTGG-3’, respectively. KpnI and XhoI enzyme sites (underline) were contained in the primers, respectively. Mutant *CYP2C19* reporter gene plasmid (2C19-Mut) was constructed as described previously by Chen et al. [14], and a substitution of 4 nucleotides of PXR response element in *CYP2C19* 5’flanking promoter region (-1876/-1880bp, tctGACCcc→tctCTGGcc) was generated using site-directed mutagenesis Kits (Stratagene, USA).

For luciferase reporter assays, HepG2 cells were seeded in 24-well plates. When at 70% confluence, the cells were transiently transfected 600ng different *CYP2C19* reporter gene plasmids with 200ng pcDNA3.1-PXR (or pcDNA3.1) and 60ng pRL-TK (Promega; USA) using Lipofectamine 2000 according to the manufacturer’s instructions for 24 hours. Then cells were incubated with 0.1% DMSO, 10μM rifampicin and NXT (150 and 250μg/mL) for another 24 hours, respectively. Once the treatment done, luciferase activities were measured by the Dual-Luciferase Reporter^™^ Assay System (Promega, USA) according to the manufacturer’s instructions. Data were normalized by Renilla luciferase activity. Each experiment was repeated at least three times in triplicate.

### Real-time quantitative PCR for CYP2C19 mRNA levels

HepG2 cells were transiently transfected with pcDNA3.1-PXR or pcDNA3.1 for 24 hours and then incubated with 0.1% DMSO, 10μM rifampicin, 150 and 250μg/mL NXT for 24 hours, respectively. Once the treatments finished, cells were washed twice with PBS and the total RNA was extracted from cells using Trizol reagent according to the manufacturer’s instructions. Then 1μg total RNA was reversed into cDNA using the first-strand cDNA synthesis Kit as follows: 42°C 2 minutes, 37°C 15 minutes and 85°C 5 seconds. The real-time quantitative PCR (qPCR) was performed using SYBR Green qPCR SuperMix Kits by LightCycler 480 (R&D, UK). The basic protocol for qPCR reactions was as follows: initial denaturation at 95°C for 30 seconds, followed by 40 cycles of denaturation at 95°C for 5 seconds, and extension at 60°C for 30 seconds. The relative expression levels of target genes were normalized to the β-actin and calculated by the 2–ΔΔCt method. Each experiment was repeated three times. The primers used for quantitative PCR were as follows: CYP2C19 F: 5’-CAACAACCCTCGGGACTTTA-3’; R: 5’-GTCTCTGTCCCAGCTCCAAG-3’; β-actin F: 5’-TGGCACCCAGCACAATGAA-3’; R: 5’-CTAAGTCATAGTCCGCCTAGA-3’.

### Whole protein extraction and Western Blotting for the CYP2C19 protein levels

HepG2 cells were transiently transfected with pcDNA3.1-PXR for 24 hours and then incubated with 0.1% DMSO, 10μM rifampicin, 150 and 250μg/mL NXT for 24 hours, respectively. Once the treatments were finished, the cells were lysed in the ice-cold RIPA buffer (Biyotime, China) containing 1% PMSF (Biyotime, China) and the concentration of the lysates was measured by BCA protein Kits according to the manufacturer’s instructions (Themo Fisher, USA).

For Western blotting analysis, proteins were separated by 10% sodium dodecyl sulfate polyacrylamide gel (SDS-PAGE) and then transferred onto PVDF membranes (Millipore, USA). After blocking in 5% non-fat milk at room temperature for 1 hour, and the membranes were incubated with primary antibodies against CYP2C19 (1:1000), and β-actin (1:5000) overnight at 4°C. The membranes were washed three times by PBS, and then were incubated with 1:5000 diluted HRP-conjugated second antibodies (1:5000) at room temperature for 2 hours. Finally the membranes were detected using the Bio-rad ChemiDoc MP System (Bio-rad; USA). β-actin levels were normalized relative abundance of the protein.

### Detection of the cell-based CYP2C19 metabolic activity

P450-Glo^™^ 2C19 Assays provide a luminescent method to measure the CYP2C19 metabolic activity. The substrates of these assays are proluciferins, derivatives of beetle luciferin. The substrates are converted by CYP2C19 enzyme to luciferin products and detected in a second reaction with the luciferin detection reagent. The amount of light produced in the second reaction is detected by a luminometer, which is proportional to the CYP activity. HepG2 cells were seeded in collagen-coated 12-well plates. When 70% confluence, HepG2 cells were transiently transfected with pcDNA3.1-PXR for 24 hours and then incubated with 0.1% DMSO, 10μM rifampicin, 150 and 250μg/mL NXT for 24 hours, respectively. The cells were washed twice by PBS and then lysed by 1×Passive Lysis Buffer (PLB) (Promega; USA). 25μL supernatant of lysate was taken to detect the CYP2C19 metabolic activity by P450-Glo^™^ 2C19 Assays (Promega; USA) according to the manufacturer’s instructions. At the same time, the concentration of the lysates was measured by BCA protein Kits (Themo Fisher, USA) to normalize the CYP2C19 activity. Each experiment was repeated at least three times in triplicate.

### Statistical analysis

All the data were presented as the mean ± S.D. from at least three independent experiments. Statistical comparisons of data were performed with one-way analysis of variance (ANOVA) followed by a post hoc test (Dunnett's test) using SPSS software (version 18.0; USA). P value < 0.05 was considered statistically significant.

## Results

### NXT enhanced the *CYP2C19* promoter activity via PXR

**Fig 1:**
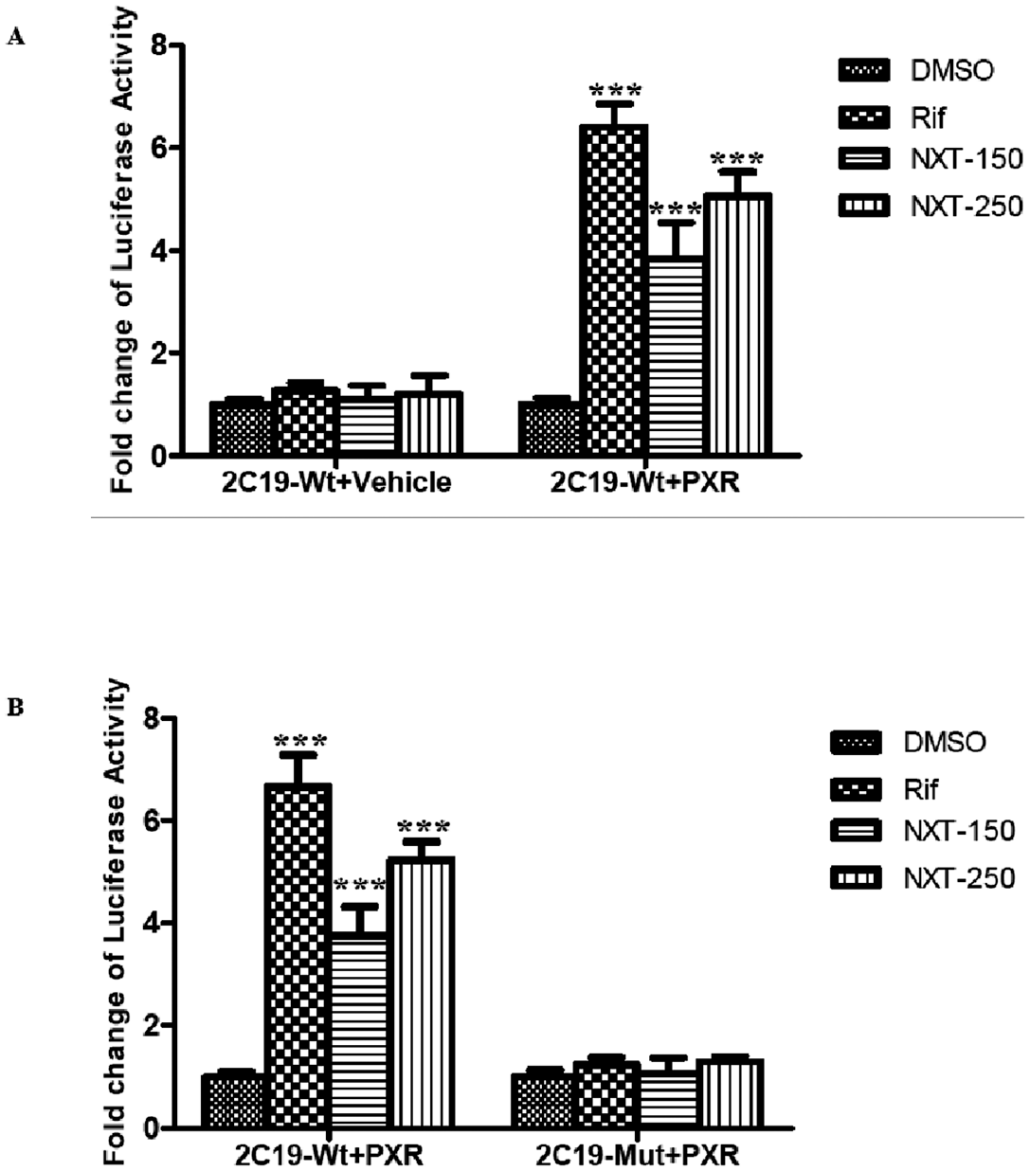
NXT activated PXR to stimulate the CYP2C19 promoter activity. HepG2 cells were transiently transfected with 2C19-Wt, 2C19-Mut and pcDNA3.1-PXR or pcDNA3.1 for 24 hours and then treated by 0.1% DMSO, 10μM rifampicin, 150 and 250μg/mL NXT for 24 hours, respectively. (A) Luciferase activity of HepG2 cells transiently transfected with 2C19-Wt and pcDNA3.1-PXR or pcDNA3.1 with treatment, was measured; (B) Luciferase activity of HepG2 cells transiently transfected with 2C19-Wt, 2C19-Mut and pcDNA3.1-PXR with treatment, was measured. Rif: rifampicin; NXT-150 and -250: 150 and 250μg/mL NXT; Vehicle: pcDNA3.1 vector; PXR: pcDNA.1-PXR expression plasmid. Data was presented as the mean±S.D from three independent experiments. ***P<0.001 vs. DMSO group.

To confirm the hypothesis that NXT regulates CYP2C19 expression at the transcriptional level via PXR, we generated wild type and the mutant *CYP2C19* 5’ flanking luciferase reporter plasmids. All these luciferase reporter plasmids were co-transfected with pcDNA3.1-PXR (or pcDNA3.1) in HepG2 cells and the luciferase activities were measured. As shown in Fig 1A, both 150 and 250μg/mL NXT as well as 10μM rifampicin significantly increased the luciferase activities of 2C19-Wt compared with the DMSO control group in HepG2-PXR cells; however, neither NXT nor rifampicin treatments caused significant difference of 2C19-Wt luciferase activities when compared with the control group in HepG2-mock cells. On the other hand, NXT and rifampicin significantly increased the luciferase activities of 2C19-Wt, but had no influence on those of 2C19-Mut compared with the control group in HepG2-PXR cells (Fig 1B).

### NXT up-regulated the CYP2C19 mRNA and protein expressions in HepG2-PXR cells

**Fig 2:**
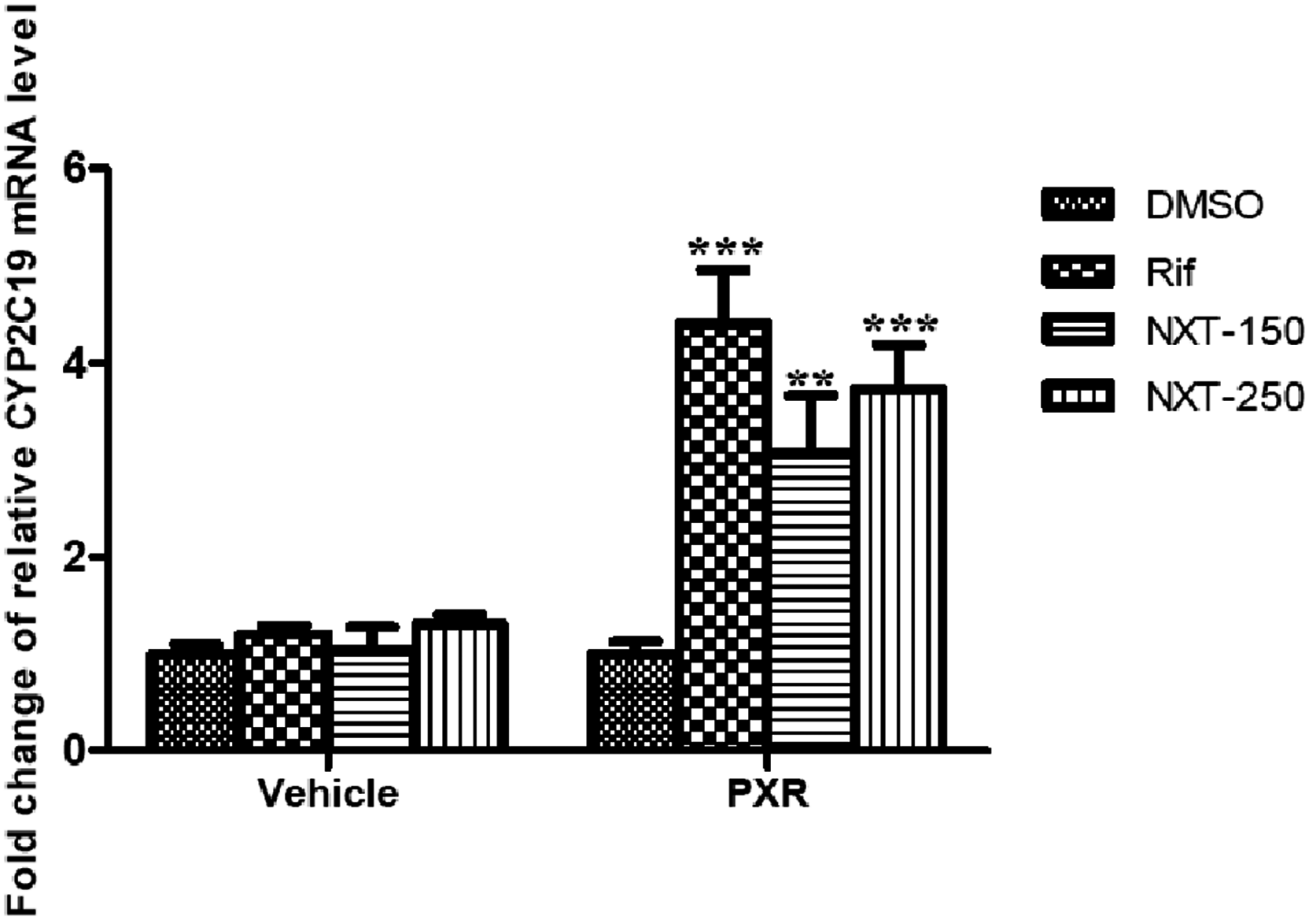
NXT inductive effects on the CYP2C19 mRNA expression in HepG2 cells. HepG2 cells were transiently transfected with pcDNA3.1-PXR or pcDNA3.1 for 24 hours and then incubated with 0.1% DMSO, 10μM rifampicin, 150 and 250μg/mL NXT for 24 hours, respectively. Rif: rifampicin; NXT-150 and -250: 150 and 250μg/mL NXT; Vehicle: pcDNA3.1 vector; PXR: pcDNA3.1-PXR expression plasmid. Data was presented as the mean±S.D from three independent experiments. ***P<0.001 vs. DMSO group.

**Fig 3:**
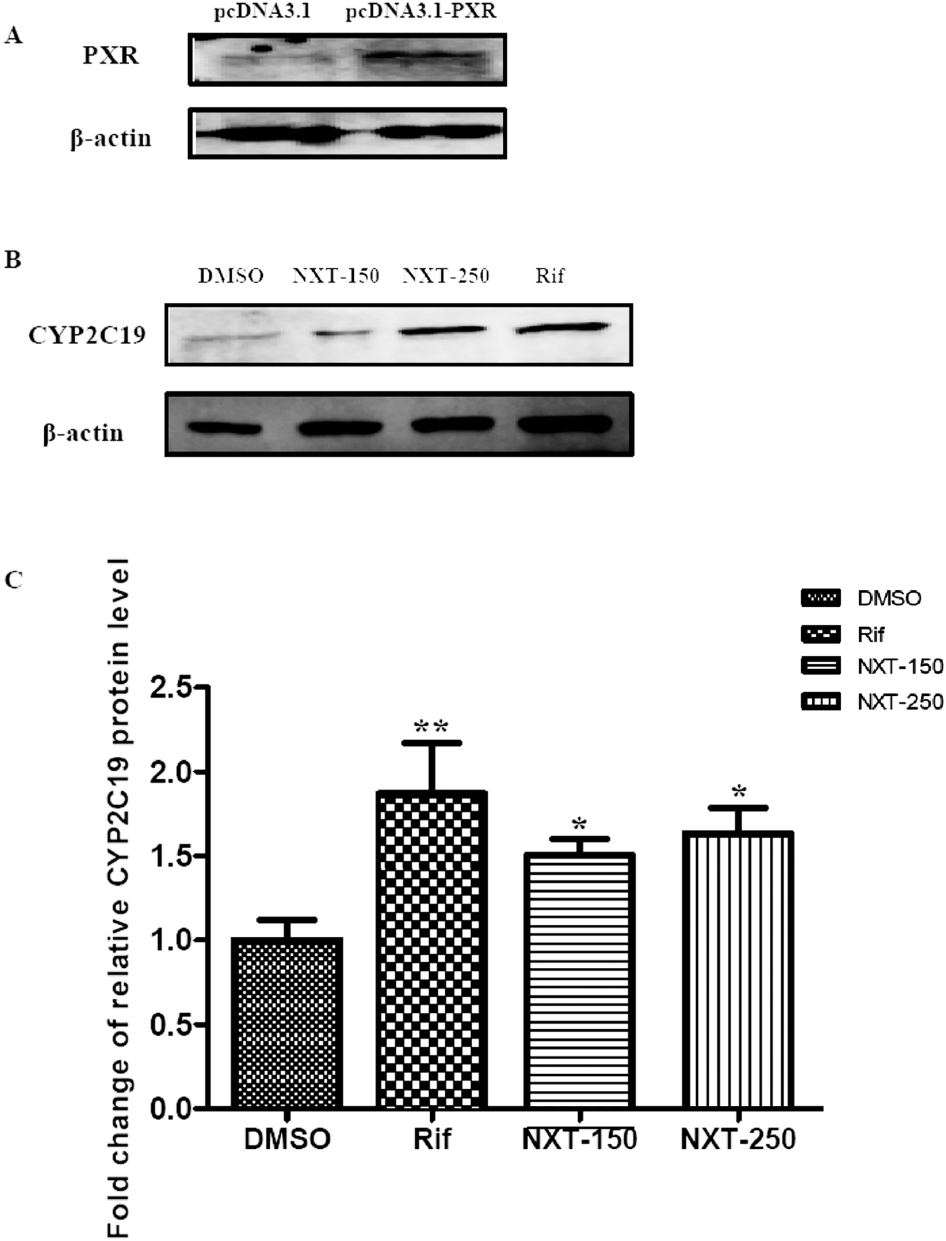
NXT induced the CYP2C19 protein expression in HepG2-PXR cells. HepG2 cells were transiently transfected with pcDNA3.1-PXR for 24 hours and then incubated with 0.1% DMSO, 10μM rifampicin, 150 and 250μg/mL NXT for 24 hours, respectively. (A) the transfection efficiency of pcDNA3.1-PXR in HepG2 cells; (B) expression of CYP2C19 protein analyzed by Western blotting; (C) accurate induction of NXT on CYP2C19 protein were quantified by densitometry. Rif: rifampicin; NXT-150 and -250: 150 and 250μg/mL NXT; Data was presented as the mean±S.D from three independent experiments. *P<0.05,**P<0.01 vs. DMSO group.

Since we found that NXT was able to increase the *CYP2C19* promoter activity in a PXR-dependent manner. We next investigated the NXT regulatory effects on the CYP2C19 mRNA and protein expression levels. As shown in Fig 2, 150 and 250μg/mL NXT treatment significantly increased the CYP2C19 mRNA expression compared with the control group in HepG2-PXR cells. Similar induction was observed in rifampicin group. On the contrary, none of NXT and rifampicin treatments caused an enhancement of CYP2C19 mRNA level compared with the control group in HepG2-mock cells. Furthermore, the results of Western blotting confirmed that NXT incubation markedly up-regulated the CYP2C19 protein expression in HepG2-PXR cells (Fig 3B and 3C). Additionally, the transfection efficiency of pcDNA3.1-PXR in HepG2 cells was also confirmed by Western blotting as shown in Fig 3A.

### NXT elevated the CYP2C19 metabolic activity in HepG2-PXR cells

**Fig 4:**
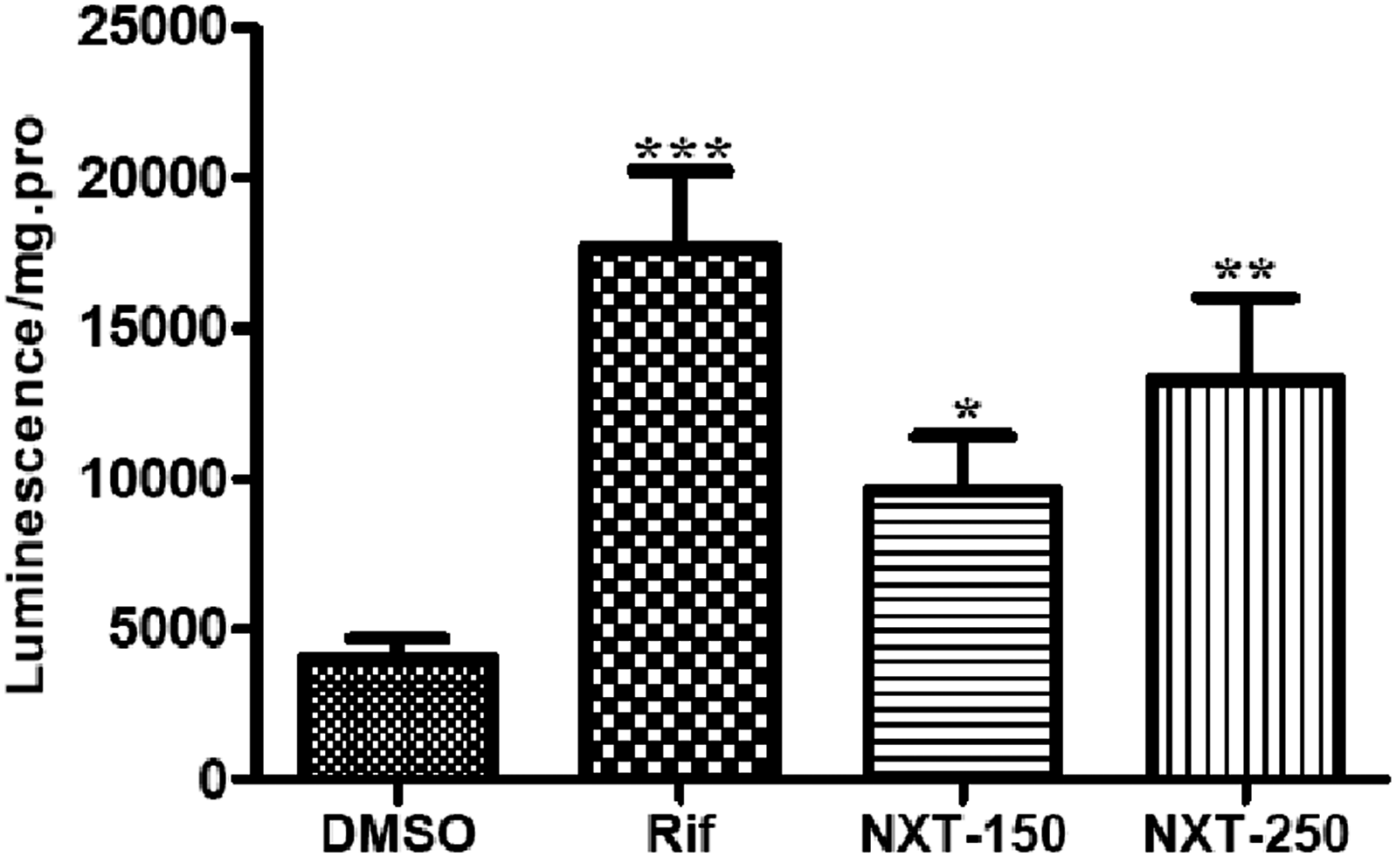
NXT elevated the CYP2C19 metabolic activity in HepG2-PXR cells. HepG2 cells were transiently transfected with pcDNA3.1-PXR for 24 hours and then incubated with 0.1% DMSO, 10μM rifampicin, 150 and 250μg/mL NXT for 24 hours respectively. Luciferin produced by CYP2C19 was detected by a luminometer and finally normalized by protein concentration. Rif: rifampicin; NXT-150 and -250: 150 and 250μg/mL NXT; Data was presented as the mean±S.D from three independent experiments. *P<0.05, **P<0.01, ***P<0.001 vs. DMSO group.

We applied P450-Glo^™^ Assay to measure the CYP2C19 metabolic ability. Luciferin produced by CYP2C19 was detected by a luminometer and finally normalized by protein concentration. As shown in Fig 4, 150 and 250μg/mL NXT treatment significantly increased the CYP2C19 metabolic activity compared with the control group in the HepG2-PXR cells. Similar enhancement was observed in rifampicin group.

## Discussion

In the present study, our data indicated that Buchang NXT was able to enhance the *CYP2C19* promoter activity and this enhancement occurred via PXR activation. Additionally, NXT could up-regulate the level of CYP2C19 mRNA and protein expressions in the HepG2-PXR cells. Finally, we observed a correspondent elevation of the CYP2C19 metabolic activity in the HepG2-PXR cells. Therefore, this is the first study to suggest a molecular mechanism for NXT-mediated inductive effects on the CYP2C19 expression and the metabolic activity, which is in favor of implication of PXR pathway.

Buchang NXT was a Chinese traditional medicine including 16 different herbs and it was identified a total of 178 components in BuChang NXT, including 21 flavones and 6 flavone glycosides, 18 phenanthraquinones, and 22 terpenoids[2, 8]. Up to now, because of it really complex compositions and intermediate metabolites, it need more experiments and analysis to find the really chemical materials of BuChang NXT that activate PXR-mediated induction of CYP2C19.

PXR belongs to the steroid/retinoid hormone receptor superfamily (NR1) of ligand-activated transcription factors. Once activated, it forms heterodimers with RXR (retinoid X receptor) and then binds to the response elements (ER6 or DR-4) in promoter domain of target genes[12]. As a key transcription regulatory factor, PXR has been implicated in the inducible expression of several DMEs and drug transporters such as CYP3A4, CYP2B6, in response to many xenobiotics[12–14]. To date, PXR-mediated CYP3A4 induction has been well studied. In addition, recent studies reported that rifampicin was also a strong inducer of CYP2C19 expression[4, 15, 16]. Chen *et al*. revealed a single PXR binding site (-1891/-1876 bp) in *CYP2C19* promoter region and CAR and PXR binding to the CYP2C19 response element in gel shifts, suggesting that PXR could bind directly to *CYP2C19* promoter to modulate its transcription and expression[15]. Although CAR could also bind to the CYP2C19 response element to modulate its transcription and expression, the expression of CAR involved in P450 regulation in HepG2 cells is minimal[19, 20]. Hence in our study, NXT induce CYP2C19 by PXR in the HepG2-PXR cells could exclude the interference of CAR. In the present study, we observed that NXT increased the *CYP2C19* promoter activity, mRNA and protein expressions only in the PXR-transfected HepG2 cells. Moreover, a markedly increase of the CYP2C19 metabolic capability was also detected using luciferin-based assays by NXT incubation. This result is consistent with our previous *in vitro* study that NXT treatment increased 4-hydroxylation of S-mephenytoin by enhanced the CYP2C19 metabolic ability[9].

Besides PXR, some other nuclear receptors or transcription factors such as CAR, GR (glucocorticoid receptor) and HNF4α (hepatic nuclear factor 4α) were also reported to be involved in modulating CYP2C19 expression. In fact, Chen *et al*. confirmed that CAR and PXR shared the same binding site in *CYP2C19* promoter region, which implied that CAR might play a role in controlling the CYP2C19 constitutive expression[15]. Moreover, a glucocorticoid-responsive element (-1750/-1736bp) was also identified[15]. As far as HNF4α, although it doesn’t bind to the PXR site directly, it could bind to its promoter and may act as co-activator to strengthen PXR/CAR effects on the *CYP2C19* transcription[21]. As we known, NXT ingredients are very complicated and some compositions of NXT such as Salvia miltiorrhiza could activate CAR to induce CYP3A4 levels[22], therefore we would like to assume that PXR plays the partial but important role in mediating NXT-induced the expression of CYP2C19, and NXT-mediated induction of CYP2C19 expression might be the cooperative results of these regulatory transcription factors, which are needed future studies to confirm.

Growing evidences illustrated that induction/inhibition of CYP enzymes is one of the risk factors for drug-drug interactions (DDIs), which have been reported to have a major clinical impact. The DDI information on CYP2C19 substrate drugs is limited. Clopidogrel is a kind of antiplatelet drugs widely used in clinics. Its active metabolite is R-130964 that is a secondary metabolite. Several DMEs including CYP3A, CYP2C19, and CYP2C9 were involved in this two-step biotransformation from clopidogrel to R-130964. Among these CYPs, CYP2C19 is responsible for about 45% production of 2-oxo-clopidogrel at the first step and nearly 20% generation of active thiol metabolite at the final step[23]. Therefore, CYP2C19*2 polymorphism, which caused a non-function protein, were reproducibly reported to be associated with significant reduction in clopidogrel active metabolite bioavailability, antiplatelet effects, and clinical outcomes[24–26]. Chinese also have a high incidence of the CYP2C19*3 null allele. Both these two variants would affect the responsiveness of clopidogrel[27]. On the other hand, CYP2C19*2 polymorphism is the predominant variation in China with a very high frequency of 41–52% in patients with coronary atherosclerotic heart disease[28]. Hence, how to overcome the low clopidogrel responsiveness in patients with CYP2C19*2 allele became an important clinical issue. Interestingly, our previous studies found that the patients undergoing PCI with CYP2C19*2 polymorphism who co-administrated clopidogrel with NXT benefit more than patients who used clopidogrel alone. In this study, we found that adjunctive NXT daily leaded to a 33% increase of residual platelet aggregation rate (Agglate) and a 34% decrease in the risk of major adverse cardiovascular events (MACEs)[11]. Now, our present study provided a reasonable explanation for this clinical DDI. NXT-induced up-regulation of CYP2C19 expression and metabolic activity is the answer for the enhanced platelet inhibitory effects of clopidogrel. Hence, it is suggested that adjunctive NXT treatment provides a novel approach to avoid clopidogrel resistance in the patients who carried CYP2C19*2 allele via increasing CYP2C19 metabolic activity.

Besides clopidogrel, it also participates in metabolism of several other important drugs such as omeprazole and lanzoprazole (proton-pump inhibitors, PPIs), mephenytoin and diazepam (antiepileptics), and citalopram (selective serotonin reuptake inhibitors). However, little is known about potential DDI between these drugs and NXT. Therefore, further studies are needed to investigate the effects of NXT on these drugs to predict impaired or increased therapeutic efficacy.

## Conclusion

In conclusion, our present study for the first time indicated that Buchang NXT capsules could activate PXR to increase the *CYP2C19* promoter transcription, mRNA and protein expression, and finally enhance the CYP2C19 metabolic activity. Our foundation could partially be the basement for DDI between some drugs or herbs and NXT.
